# Delivering the message: mRNA replacement reaches the liver in Niemann-Pick type C1 disease

**DOI:** 10.1016/j.omtn.2026.103109

**Published:** 2026-10-07

**Authors:** Laura Lentini, Emanuele Vitale

**Affiliations:** 1 Department of Biological, Chemical and Pharmaceutical Sciences and Technologies, University of Palermo, Palermo, Italy

## Main text

Niemann-Pick type C1 (NPC1) disease is an autosomal recessive lysosomal storage disorder affecting both neuronal and peripheral tissues. Several NPC1 mutations impair cholesterol export from late endosomes and lysosomes, leading to lipid accumulation, impaired autophagy, mitochondrial stress, inflammation, and progressive injury in the liver and central nervous system. Although the conditions can be severe, available therapeutic options remain limited. In this issue of *Molecular Therapy Nucleic Acids*, Koufer and colleagues reported results from an *in vivo* evaluation of an mRNA-based NPC1 replacement therapy. In detail, using lipid nanoparticles (LNPs) carrying a codon-optimized murine Npc1 mRNA (Co-Npc1:LNP), they restored a correctly folded and lysosome-targeted NPC1^1^ (Figure 1). This study shows that temporary expression of a large lysosomal membrane protein can rapidly improve systemic defects. However, the durability of the effects, repeat dosing, and delivery to the nervous system must be evaluated.^1^

**Figure 1:**
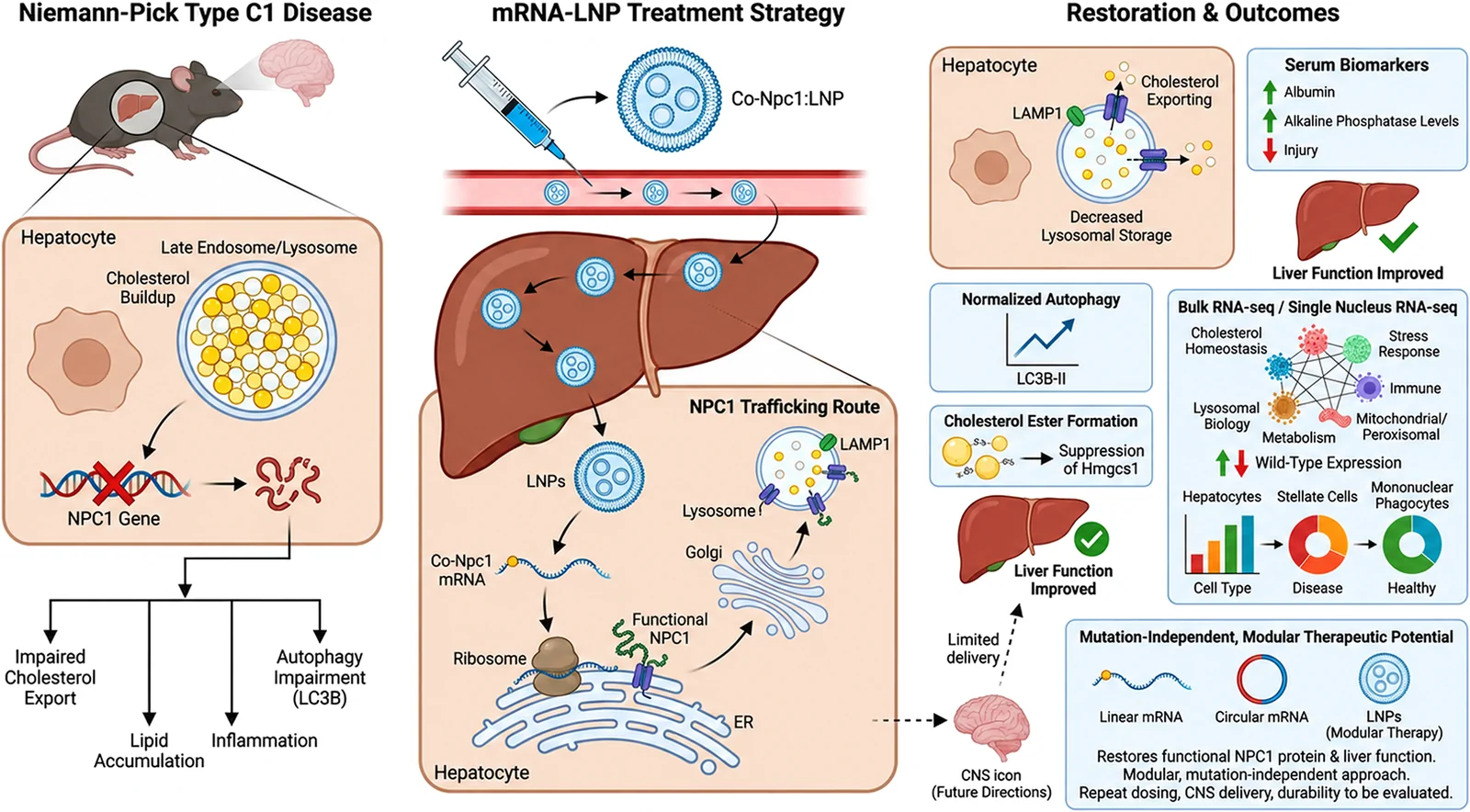
mRNA-LNP-mediated NPC1 replacement restores cholesterol homeostasis and liver function in Niemann-Pick Type C1 disease (prepared by FigureLabs)

NPC1 disorder is caused by biallelic loss-of-function variants in the NPC1 gene, which encodes a 1,278-amino-acid glycoprotein normally residing in late endosomes which interacts with lysosomes and the *trans*-Golgi network^2^^,^^3^ More than 300 pathogenic variants have been described, and the clinical phenotype ranges from neonatal cholestatic liver disease to later-onset neurodegeneration. The liver is one of the most targeted organs of the disease; in particular, hepatic disease can dominate in neonates and children; furthermore, a subset of infants with hepatomegaly develops fatal liver failure.^2^ As described in the literature, the defective intracellular cholesterol trafficking leads to multiple interconnected abnormalities; thus, the mRNA-based NPC1 replacement strategy could act upstream of the lysosomal, autophagic, metabolic, and inflammatory consequences, directly addressing the primary molecular defect.^2^

The use of messenger RNA highlighted good efficacy as a new category for the treatment of various diseases, allowing a form of protein replacement without genomic integration. The development of chemical modification and LNP encapsulation enriched this strategy, reducing innate sensing, protecting the transcript, and promoting cytosolic delivery; furthermore, the known accumulation of LNPs in the liver makes this strategy particularly suitable for treating liver diseases.^4^ The Niemann-Pick type C1 disease also shows another complex challenge; in fact, NPC1 protein is a large, heavily glycosylated, multipass protein that must fold in the endoplasmic reticulum (ER), traverse the Golgi, and reach the lysosomal membrane in the correct orientation.^2^ Thus, the codon optimization and the administration mediated by LNPs may increase protein output, but it must not compromise folding or trafficking, so Koufer and colleagues focused not only on NPC1 expression, but whether an exogenous transcript can recreate the full cellular itinerary of a functional lysosomal transporter.^1^

The development of an Npc1 and a codon-optimized Npc1 mRNAs and their LNP encapsulation was essential to study, as a first step, the restoration of protein production in NPC1-deficient HeLa cells. The codon optimization did not alter mRNA stability, or transcript stabilization, but it increased protein abundance and prolonged functional output. Crucially, the resulting protein was predominantly resistant to endoglycosidase H and colocalized with LAMP1, supporting maturation through the Golgi and arrival at lysosomes. Furthermore, the employment of Co-Npc1:LNP reduced filipin-positive cholesterol storage, increased cholesteryl ester formation, and normalized the autophagy marker LC3B-II for longer than the non-optimized construct. These results allow Koufer and colleagues to distinguish the functional replacement from the sample protein expression, leading to the selection of Co-Npc1:LNP for *in vivo* testing.

Luciferase studies using a 1 mg/kg dose of LNPs carrying firefly luciferase (LUC) mRNA construct, administered intravenously to Npc1-null mice, confirmed that LNP delivery was concentrated in the liver. Npc1-null mice received a single intravenous injection of 1 mg/kg Co-Npc1:LNP, and after 48 h, hepatic NPC1 protein was restored to below wild-type abundance but retained the glycosylation pattern expected of successfully trafficked protein. In addition, the mice showed an endo H-resistant NPC1 protein, confirming the effective escape of LNP-encoded NPC1 from the ER and its successful trafficking to lysosomes *in vivo*.^1^ As already demonstrated *in vitro* and *in vivo*, LC3B-II levels were normalized after Co-Npc1:LNP administration, while lipidomic analyses showed increased cholesteryl ester species and suppression of Hmgcs1, confirming the restoration of cholesterol trafficking and the correction of Niemann-Pick type C1 defects. Of note is that the control GFP:LNP-treated mice increased unesterified cholesterol and the synthetic lipid SM-102, whereas Co-Npc1:LNP-treated mice revealed esterified cholesterol and SM-102 increase but not unesterified cholesterol, suggesting that LNP introduction causes the accumulation of synthetic lipid components while the NPC1 protein recovery leads to the production of esterified cholesterol pools, consistent with the restoration of intracellular trafficking and processing.^1^ Lastly, the analysis of serum biomarkers of liver injury, such as albumin and alkaline phosphatase levels, suggests an improvement in liver function.^1^

Furthermore, the rationale of the study by Koufer and colleagues extends beyond investigating the recovery of NPC1 protein expression, focusing also on the biological relationships among genes that share similar regulatory pathways to determine how the treatment affects interconnected biological processes. For this aim, bulk RNA sequencing (RNA-seq), single-nucleus RNA-seq, the network-based MENTOR framework, and the MENTOR-Interpretation Agent tool were employed for the analysis. All these analyses showed 714 genes altered between therapeutic and control LNP treatment, of which 645 changed in the direction opposite to the disease signature. In addition, results demonstrated that functional modules identified by the MENTOR system and associated with cholesterol homeostasis, lysosomal biology, stress responses, energy metabolism, immune activity, and mitochondrial and peroxisomal processes shifted toward wild-type expression. Finally, single-nucleus RNA-seq analyses showed cell type-specific transcriptional changes, confirming the bulk RNA-seq analyses after Co-Npc1:LNP treatment, demonstrating that hepatocytes, hepatic stellate cells, and mononuclear phagocytes show transcriptional shifts toward a wild-type phenotype, suggesting more than a single biomarker correction but a therapeutic option.

Although the liver is one of the organ targets of the disease, NPC1 disorder is ultimately dominated in many patients by progressive neurological deterioration; however, conventionally administered intravenous LNPs preferentially target the liver and do not efficiently cross the blood-brain barrier.^5^ However, hepatic therapy may represent a clinically meaningful option for neonatal disease by stabilizing vulnerable infants or complementing a brain-directed therapeutic approach. In future, repeat-dose studies must also define pharmacokinetics, the minimum NPC1 activity required for benefit, and immune responses to the LNP and newly expressed protein. In conclusion, the short persistence of the linear mRNA also motivates evaluation of improved untranslated regions, circular RNA, or self-amplifying architectures, provided expression remains controllable.^1^^,^^5^^,^^6^ Koufer and colleagues demonstrate that transient mRNA delivery can successfully address proper NPC1 production inside the cell, compensating for disease-related pathological alterations. The most important conclusion is related not only to simple protein recovery but also highlights that a complex lysosomal membrane protein can be replaced without editing the genome. Crucially, if repeat dosing demonstrates safety without toxicity, and the delivery can be expanded beyond the liver, mRNA replacement could become a modular, mutation-independent strategy not only for NPC1 disease but also for other disorders. To date, Co-Npc1:LNP represents a promising strategy against NPC1 disease that highlights the therapeutic potential of mRNA replacement, providing a strong foundation for its translation into durable clinical benefit.

## Author contributions

L.L. and E.V. wrote the commentary.

## Declaration of interests

The authors declare no competing interests.

## Declaration of generative AI and AI-assisted technologies in the writing process

During the preparation of this commentary, the authors used Grammarly for the editing and FigureLabs to produce the figure.
